# The Joint Effects of Loneliness and Depression on Mortality: Does Gender Matter?

**DOI:** 10.1093/geroni/igaa057.1034

**Published:** 2020-12-16

**Authors:** Ted Kheng Siang Ng, Abhijit Visaria, Angelique W M Chan, Kheng Siang Ted Ng

**Affiliations:** 1 DUKE-National University of Singapore, Singapore, Singapore; 2 Duke-NUS Medical School, Singapore, Singapore; 3 Yong Loo Lin School of Medicine, National University of Singapore, Singapore

## Abstract

Loneliness and depression are both associated with an increased risk of all-cause mortality among older adults. However, the evidence on the joint effect of loneliness and depression is scarce. Furthermore, previous research has rarely examined the modifying effects of gender. We investigated these questions using the Panel on Health and Aging of Singaporean Elderly, a nationally-representative cohort study of community-dwelling older Singaporean adults aged 60 and above, conducted in 2009 with two follow-up waves in 2011 and 2015 (N=4536). We operationalized six groups based on three categories of loneliness measured using the 3-item University of California, Los Angeles (UCLA) loneliness scale: always lonely, sometimes lonely, and never lonely; Two categories of depressive symptom scores were measured using the 11-item Center for Epidemiologic Studies Depression Scale (CES-D) scale: depressed and not depressed. Cox proportional hazards models were employed to estimate the mortality risks for each group, with an extensive set of covariates. Due to significant differences in the prevalence of loneliness and depression in different genders, we conducted gender-stratified analyses. Compared to being not depressed and never lonely, women who were depressed and sometimes lonely and who were not depressed but always lonely had a higher mortality risk. Men who were not depressed but sometimes lonely had a higher mortality risk. We conclude that loneliness appears to be the predominant construct in conferring excess mortality risk. Health policies and interventions addressing the factors common and unique to each gender may improve psychological well-being at older ages, thereby extending the lifespan.

